# Contrast and Phase Combination in Binocular Vision

**DOI:** 10.1371/journal.pone.0015075

**Published:** 2010-12-09

**Authors:** Chang-Bing Huang, Jiawei Zhou, Yifeng Zhou, Zhong-Lin Lu

**Affiliations:** 1 Laboratory of Brain Processes (LOBES), Departments of Psychology and Biomedical Engineering, Neuroscience Graduate Program, University of Southern California, Los Angeles, California, United States of America; 2 Hefei National Laboratory for Physical Sciences at Microscale and School of Life Science, University of Science and Technology of China, Hefei, People's Republic of China; University of Dayton, United States of America

## Abstract

**Background:**

How the visual system combines information from the two eyes to form a unitary binocular representation of the external world is a fundamental question in vision science that has been the focus of many psychophysical and physiological investigations. Ding & Sperling (2006) measured perceived phase of the cyclopean image, and developed a binocular combination model in which each eye exerts gain control on the other eye's signal and over the other eye's gain control. Critically, the relative phase of the monocular sine-waves plays a central role.

**Methodology/Principal Findings:**

We used the Ding-Sperling paradigm but measured both the perceived contrast and phase of cyclopean images in three hundred and eighty combinations of base contrast, interocular contrast ratio, eye origin of the probe, and interocular phase difference. We found that the perceived contrast of the cyclopean image was independent of the relative phase of the two monocular gratings, although the perceived phase depended on the relative phase and contrast ratio of the monocular images. We developed a new multi-pathway contrast-gain control model (MCM) that elaborates the Ding-Sperling binocular combination model in two ways: (1) phase and contrast of the cyclopean images are computed in separate pathways, although with shared cross-eye contrast-gain control; and (2) phase-independent local energy from the two monocular images are used in binocular contrast combination. With three free parameters, the model yielded an excellent account of data from all the experimental conditions.

**Conclusions/Significance:**

Binocular phase combination depends on the relative phase and contrast ratio of the monocular images but binocular contrast combination is phase-invariant. Our findings suggest the involvement of at least two separate pathways in binocular combination.

## Introduction

We see the world with two eyes. It is remarkable that, most of the time, we perceive a single image of the world despite each eye having its own unique retinal image [1]. How unity of vision is achieved by binocular combination is a fundamental question in vision science [2], [3]. A large number of psychophysical and physiological studies have investigated how two identical monocular spatial patterns combine to generate a single cyclopean image [4]–[12], two slightly different monocular patterns fuse to generate depth perception [13]–[16], or two very different monocular patterns give rise to binocular rivalry [17]–[23]. However, until recently, one critical aspect of binocular combination has been largely neglected, that is, how two different monocular spatial patterns are combined to generate a single cyclopean percept, although that is perhaps the most common situation when we perceive the external visual world with two eyes.

Ding and Sperling [24] were the first to measure the appearance of cyclopean image resulted from binocular combination of sine-wave gratings with identical frequency but different phases and contrasts in the two eyes, although contrast discrimination thresholds have been investigated in some previous studies [25], [26]. The perceived cyclopean image is a sine-wave grating, whose perceived phase is determined by the contrast ratio and phase difference between the monocular inputs. They proposed a contrast-gain control model that has been very successful in modelling phase perception in binocular vision in normal vision [24], [27], and extended by us to successfully model binocular phase combination in amblyopic vision [27]. Here, we attempt to develop a more complete model of binocular combination, by investigating how binocular combination generates the perception of *both* phase and contrast from different monocular spatial patterns. We found that, *surprisingly*, the perceived contrast of cyclopean images was independent of the relative phase of the monocular sine-wave gratings, although the perceived phase of the cyclopean images depended on the relative phase and contrast ratio of the monocular images. We propose a new multi-pathway contrast-gain control model (MCM) of binocular combination.

**Figure 1:**
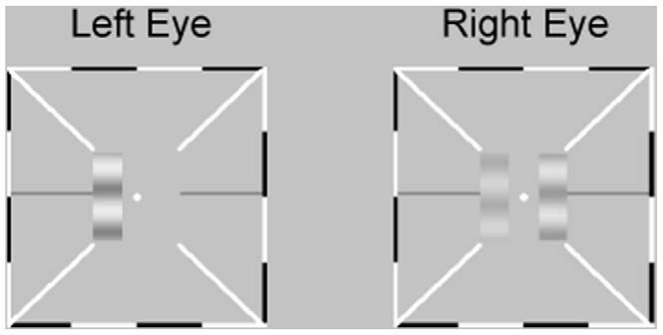
Stimulus display. The two panels were delivered to the left and right eyes using a stereoscope. The two test gratings on the left in the two eyes' views, differing in contrast and phase, are combined via stereoscope. Observers adjusted the contrast and phase of the monocular probe grating to match those of the cyclopean image.

We elaborated the Ding-Sperling binocular combination paradigm to measure both the perceived phase and contrast of the cyclopean percept. A stereoscope was used to present three sine-wave gratings to the observer in each trial (Figure 1): two test gratings on the left of fixation in both eyes and a monocular probe grating presented to one eye. Binocular presentation of the two test gratings, with proportional contrasts and different phases, produced a single cyclopean percept. Four observers adjusted the phase and the contrast of the probe grating to match the perceived phase and contrast of the cyclopean image. The phase and contrast of the cyclopean sine-wave precept were measured as functions of the base contrast level, the contrast ratio between the two eyes, the phase difference between the test gratings, and the dichoptic configuration (+ and – phase shifts in the left and right eyes, and vice versa), for a total of 216 (3 base contrasts ×6 interocular ratios×3 phase differences ×2 probe eye ×2 configurations) and 144 (3×6×2×2×2) conditions for perceived contrast and phase, respectively.

## Results

**Figure 2:**
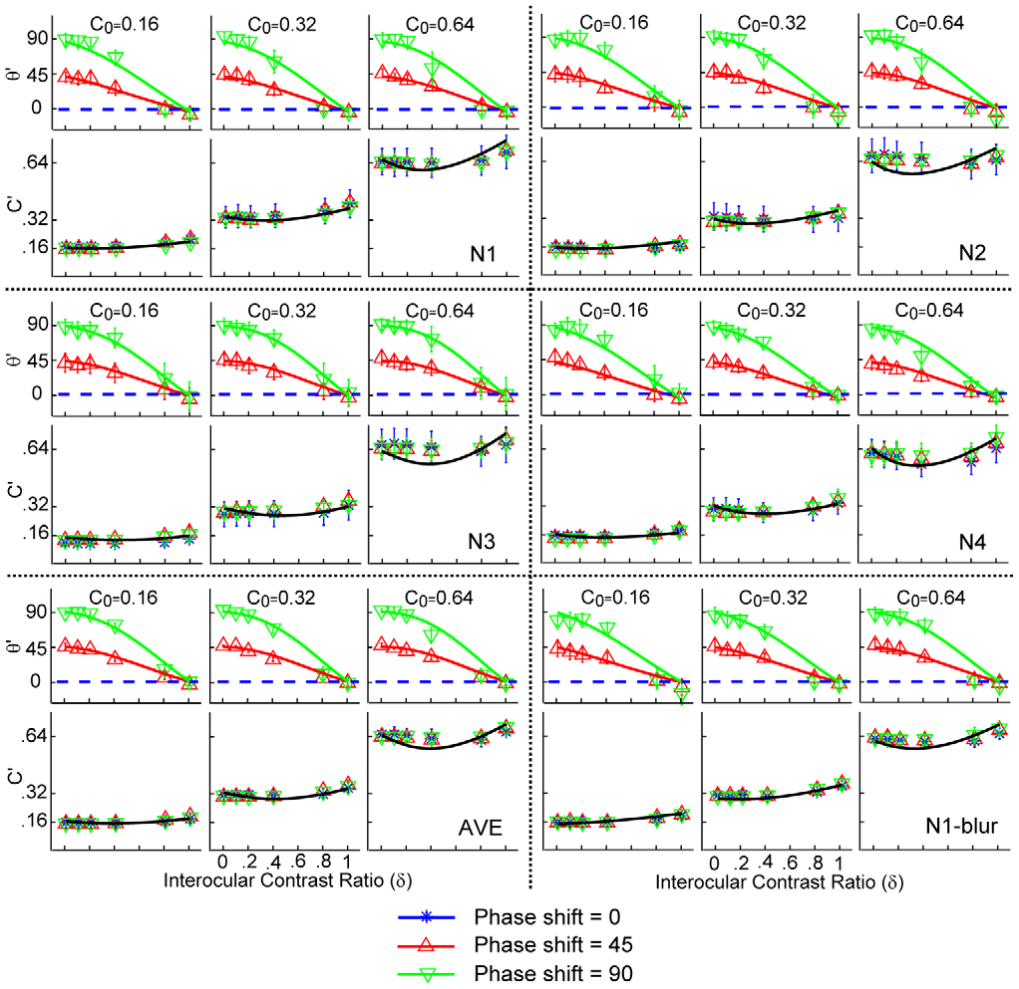
Perceived contrast and phase of the cyclopean images. Data from different base contrast conditions are shown in separate panels. For each observer, data from the three base contrast conditions are shown in three columns. Within each column, the upper row shows the perceived phase (in degrees) and the lower row shows the perceived contrast, both as a function of the interocular contrast ratio. Different colors denote different phase shift conditions: blue asterisk for 0 degree, red upward-pointing triangle for 45 degrees, and green downward-pointing triangle for 90 degrees. Subjects only performed the phase matching task in the 45 and 90 degree conditions. The blue dashed line indicates expected output with zero phase difference. Error bars represent standard deviations.

The perceived phase 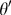 of the cyclopean images is plotted as a function of the contrast ratio between the test gratings in the two eyes in Figure 2, with data from three base contrast conditions in separate panels. The perceived phase of the cyclopean image depended strongly on interocular contrast ratios (F(5,15) = 397.95, p<0.001), but not on base contrast (F(2,6) = 0.47, p>0.10), nor on the probe eye condition (F(1,3) = 0.46, p>0.10). Data from the two probe eye conditions were pooled in Figure 2 and in subsequent analyses. Increasing the interocular contrast ratio from 0 (a single test grating in one eye) to 1.0 (two test gratings with equal contrast in two eyes), produced a monotonic decrease of perceived phase from either 45 or 90 deg to approximately 0 deg, for the 45 and 90 deg phase shift conditions, respectively. Because the gratings in the two eyes were always phase-shifted with equal magnitude in opposite directions, the phase of the cyclopean percept should be 0 deg when the two gratings generate equal internal representations in binocular phase combination. Consistent with previous reports [24], [27], our results suggest that signals from the two eyes contribute almost equally in binocular phase combination.

In Figure 2, the perceived cyclopean contrast, 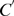, is plotted as a function of interocular contrast ratio, with data from three base contrast conditions in separate panels. Surprisingly, data from the three phase-shift conditions virtually overlapped, i.e., the perceived contrast of the cyclopean image did not depend on the phase difference of the two monocular test sine-wave gratings (F(2,6) = 0.07, p>0.50) in all three base contrast conditions (F(4,12) = 0.32, p>0.50) for all four observers. The two dichoptic stimulus' configurations yielded essentially identical estimates (F(1,3) = 2.183, p>0.10). The probe eye condition had a significant (F(1,3) = 28.75, p = 0.013) but small effect: the mean ratio of the perceived contrast of the cyclopean grating measured with the probe in the dominant (left) and non-dominant (right) eye is 1.06±0.03, indicating a small imbalance of the two eyes in binocular contrast combination (i.e., one eye is slightly more dominant than the other). We pooled the data in the two dichoptic configurations and probe eye conditions in subsequent analyses.

**Figure 3:**
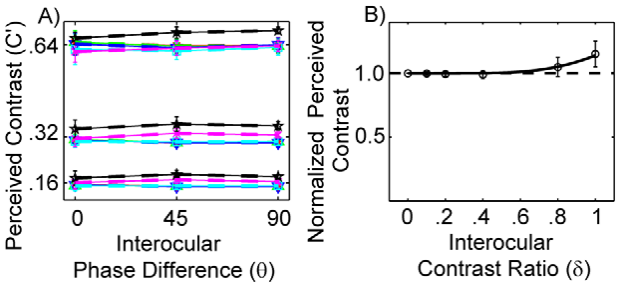
Phase-invariance and binocular advantage of contrast combination. (A) Average perceived contrast (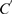) of the cyclopean images versus interocular phase difference (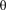) in three different base contrast and six contrast ratio conditions. Red asterisk, green upward-pointing triangle, blue downward-pointing triangle, cyan square, magenta cross and black five-pointed star represent data from the six contrast ratio (δ = 0, 0.1, 0.2, 0.4, 0.8 and 1.0) conditions. (B) Normalized perceived contrast as a function of interocular contrast ratio. The red curve represents the fit with the equation 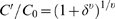 with υ = 6.07.

**Figure 4:**
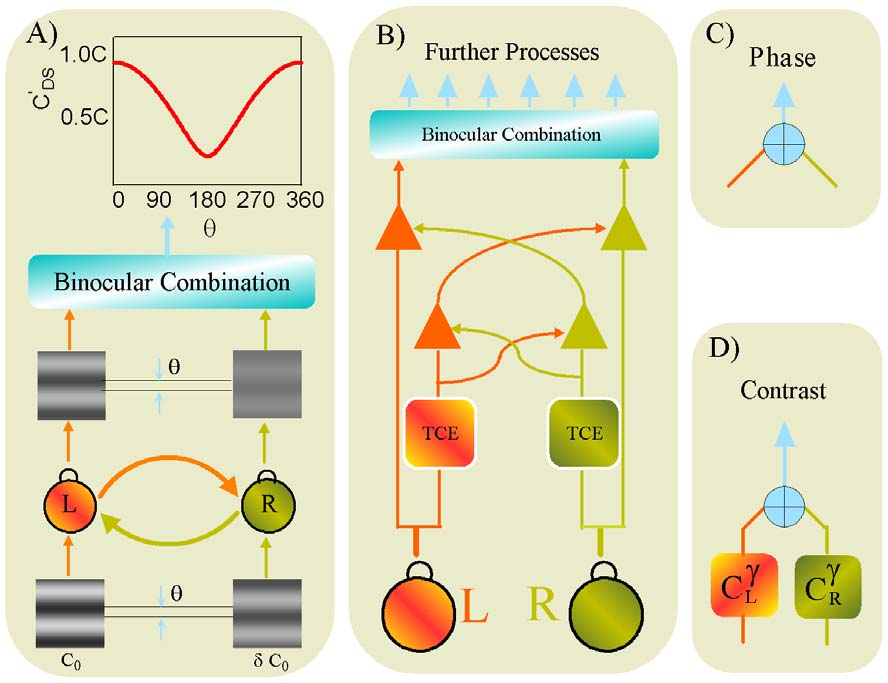
Multi-pathway contrast-gain control model (MCM) of binocular combination. (A) Perceived contrast of the cyclopean images predicted by phase-dependent contrast binocular combination models (e.g., the Ding-Sperling model). (B,C,D) Schematic diagram of the multi-pathway contrast-gain control model (MCM) of binocular combination. Signals first go through double interocular contrast gain control (B), in which each eye exerts gain control on the other eye's signal in proportion to its own signal contrast energy, and also gain-controls over the other eye's gain control. The multi-pathway contrast-gain control model (MCM) of binocular combination elaborates the Ding-Sperling binocular combination model in two ways: (1) Phase and contrast of the cyclopean images are computed in separate pathways (C and D), although with shared cross-eye contrast-gain control; and (2) phase-independent local energy from the two monocular images are used in contrast combination.

To better illustrate the phase-independent property of perceived contrast in binocular contrast combination, we re-plotted the average perceived contrast of the four observers as functions of the phase-shift between the two monocular test gratings (Figure 3A). Indeed, the perceived contrast versus phase curves are flat in all three conditions. Averaged over interocular contrast ratios and observers, the perceived contrast of the cyclopean image from binocular combination of two gratings with 0, 45 and 90 deg of phase shifts was 0.16±0.01 (mean±s.d.), 0.16±0.02 and 0.16±0.01 when the base contrast was 0.16; 0.32±0.01, 0.32±0.03 and 0.32±0.02 when the base contrast was 0.32; and 0.64±0.02, 0.64±0.02 and 0.64±0.02 when the base contrast was 0.64 (Figure 3A). The pattern of results contradicts phase-dependent models of binocular contrast combination. Regardless of the detailed computations in binocular contrast combination, phase dependent models of binocular contrast combination predict a factor of 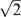 in perceived contrast between the 0 and 90 deg phase-shift conditions when the effective contrasts of the two monocular images are equal (Figure 4A).

We can also evaluate whether two eyes are “better” than one. The average (across base contrast levels, phase shifts between monocular images, and subjects) normalized perceived contrast, defined as the contrast of the matched probe grating divided by the base contrast of each condition, is plotted in Figure 3B as a function of interocular contrast ratio between the monocular images. The normalized ratio is very close to 1.0 for contrast ratios up to 0.8– in other words, the perceived contrast of the cyclopean image was equal to the higher contrast of the two monocular images; the eye with lower contrast didn't contribute much in perceived contrast. The normalized ratio is 1.05 and 1.15 when the contrast ratio is 0.8 and 1.0, respectively, that is, two eyes were better than one only when the contrasts in the two eyes are close. The results suggest strong interocular contrast gain control in binocular combination. We fitted a simple model 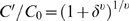 to the normalized ratios, and found that υ = 6.07 provided the best fit.

To control for potential contamination of high spatial frequencies presented in the edges of the sine-wave gratings [28], we blurred the edges of the sine-wave gratings and re-tested one of our observers (N1). The results with and without edge-blur are essentially the same (Figure 2), indicating that our original results were not due to high spatial frequency contaminations.

In summary, we found that the perceived contrast of the cyclopean images was independent of the relative phase of the monocular sine-wave gratings, although the perceived phase of the cyclopean images depended on the relative phase and contrast ratio of the monocular images. The findings of contrast-dependent phase combination and phase-independent contrast combination suggest that at least two separate pathways are involved in binocular combination. The new results require a reconsideration of existing binocular combination models.

We elaborated the Ding-Sperling model, originally developed and successfully applied to model binocular phase combination, to develop a new multi-pathway contrast-gain control model (MCM, Figures 4 and Text S1). In the MCM, the phase and contrast of the cyclopean percept are computed in separate pathways [29] after double interocular contrast gain-control [30]–[32]. Like Ding and Sperling [24], the MCM computes the perceived phase of the cyclopean images by summing the outputs from double interocular contrast-gain control, i.e., each eye exerts gain control over the other eye's signal in proportion to its own signal contrast energy, and the gain control that the other eye exerts. The phase information is kept in the contrast gain control process, and the model extracts phase-independent contrast energy from the two monocular images, and combines them using a power law [5], [7] to compute perceived contrast of the cyclopean images. In total, the MCM has three free parameters: the nonlinearity factor (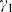) in the contrast gain control process, the gain control efficiency of the signal strength (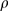) and the exponent that controls the power-law summation (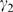).

The MCM model successfully accounted for 99.4% and 98.7% of the variance in perceived phase and contrast of the cyclopean images for the average observer, with goodness of fit ranging from 98.9% to 99.6% for binocular phase combination and 96.5% to 99.3% for contrast combination for individual observers (Table 1 and Figure 2). The MCM is also superior to the Ding-Sperling model that predicts phase-dependent binocular contrast combination in all observers and their average for binocular contrast combination (p<0.001). The parameters of the best fitting model are listed in Table 1.

**Table 1:** Parameters of the best fitting model.

	γ_1_	γ_2_	ρ	*r^2^__cont_*	*F*(1,87)	*r^2^__phase_*	*F*(1,87)
AVE	1.11	0.90	76.51	0.99	957.38**	0.99	127.48**
N1	0.95	0.89	14.28	0.96	296.20**	0.99	17.82**
N2	1.00	0.92	23.12	0.97	151.89**	0.99	11.93**
N3	1.13	0.88	136.72	0.99	206.03**	0.99	0.57
N4	0.86	0.93	42.77	0.99	316.36**	0.99	27.21**
N1_blur	1.10	0.93	8.49	0.99	303.42**	0.96	13.49**

** , p<0.001.

## Discussion

In this study, we elaborated the Ding-Sperling paradigm to measure both the perceived phase and contrast of the cyclopean images generated by binocular combination of two monocular sine-wave gratings. We found that the perceived contrast of cyclopean images was independent of the relative phase of the monocular sine-wave gratings, although the perceived phase of the cyclopean images depended on the relative phase and contrast ratio of the monocular images. The findings of contrast-dependent phase combination and phase-independent contrast combination suggest the involvement of at least two separate pathways in binocular combination. We developed a multi-pathway contrast-gain control model (MCM) of binocular combination to account for our empirical results.

Most previous studies on binocular combination have investigated how two identical monocular images combine [2], [5], [7], [8]. Two popular models, probability summation [8] and quadratic summation [5], [9], have been proposed for binocular combination of contrast signals near threshold. Power summation [7], two-stage gain control [2], twin summation [10], and binocular normalization [33], have been proposed for supra-threshold binocular contrast combination. Because the relative phase between monocular images was set to zero in these studies, the phase information was absent in these models. On the other hand, when the relative phase of the monocular images is zero, the contrast combination branch of the MCM is very similar to those earlier models.

The idea of multiple pathways for binocular combination is also consistent with physiological findings. There are both simple and complex cells in primary visual cortex [29]. Whereas simple cells, which receive inputs from lateral geniculate nuclei, respond to visual stimuli in a roughly linear manner and are phase sensitive; complex cells, which pool responses of multiple simple cells through recurrent networks [34], respond to visual stimuli in a highly nonlinear manner and are phase invariant. Phase sensitive combination of outputs of simple cells is important for stereopsis, where phase-independent combination could result from combination of outputs of complex cells [35], [36]. Models of visual cortex have also specified edge sensitive and surface sensitive computations [37]–[39].

In this study, we investigated the appearance of the cyclopean images from supra-threshold binocular contrast combination of monocular sine-wave gratings with relative phase-shifts up to 90 deg. We didn't study larger phase difference because of binocular rivalry in those conditions. It would be necessary to further test if the MCM can be used to model phenomena in near threshold conditions because appearance and contrast detection/discrimination may be computed in separate pathways [40]. For example, Blakemore & Hague [15] found that two in-phase sinusoidal gratings in the two eyes were more readily detected than out-of-phase gratings, even though the magnitude of detect-ability improvement was small. Others also documented that binocular advantage is higher for the in-phase than the out-of-phase condition in contrast discrimination of supra-threshold gratings [25], [26]. The phase-dependent effect in binocular detection is reversed and enlarged when gratings were displayed in either narrowband [41] or broadband [42] visual masking noise. It would also be interesting to investigate binocular combination in external noise [24], [43].

Our results support at least two separate pathways, phase and contrast combination, in the MCM. The MCM has already provided an important theoretical framework in elucidating binocular deficits in amblyopia [44]. In future studies, we will examine other phenomena in binocular interaction, e.g., stereo vision [13], binocular rivalry [18], [21], [22], and interocular masking [17], to test and specify additional pathways of binocular combination.

## Materials and Methods

### Observers/Ethics Statement

Four adult observers (22-28 yrs old), with normal or corrected-to-normal vision and naïve to the purpose of the experiment, participated in the study with written informed consent. The research protocol was approved by the Ethics Committee of the University of Science and Technology of China.

### Apparatus

All stimuli were generated by a PC computer running Matlab (MathWorks, Inc.) with PsychToolBox 2.54 extensions [45], [46], and presented on a Sony G220 Triniton monitor with a 1600×1200 resolution and a 75 Hz vertical refresh rate. A special circuit (http://lobes.usc.edu/videoswitcher.html) was used to combine two 8-bit output channels of the video card to yield 14-bit gray-scale levels [47] that was then scaled linearly using a psychophysical procedure [47]. A modified Helioth-Wheatstone stereoscope [1], [48] was used to present the dichoptic images to the two eyes. The stereoscope and a chin rest were mounted on a table with a 105 cm total optical path.

### Stimuli

Stimuli were three horizontal sine-wave gratings, each subtending 0.67×2 deg^2^ (Figure 1). The luminance profiles of the two test gratings on the left in the two eyes' views are: 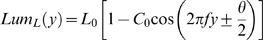
(1)

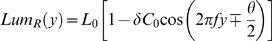
(2)where 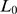 = 31.2 cd/m^2^ is the background luminance, 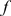 = 1 c/deg is the spatial frequency of the gratings, 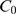 is the base contrast, and 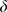 is the interocular contrast ratio. The two gratings are phase-shifted in opposite directions by 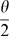, with a total phase difference of 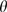. The two monocular test sine-wave gratings were viewed through the stereoscope to generate a single cyclopean sine-wave grating. Three base contrast levels (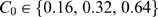), six interocular contrast ratios (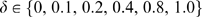), and three phase-shift differences (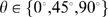), were tested.

The luminance profile of the probe sine-wave grating on the right visual field in one eye is:
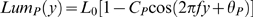
(3)where 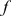 = 1 c/deg is the same as that of the test gratings, and both the contrast 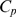
and phase 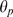 of the probe grating were adjusted by the observer to match those of the cyclopean image on the left side of the display. The probe grating was presented either to the left or the right eye.

To control for potential contamination from the high spatial frequencies presented in the edges of the sine-wave gratings, we re-tested observer N1 with edge-blurred sine-wave gratings. A 0.53 deg half-Gaussian envelope (σ = 0.1 deg) was applied to the left and right edges of the gratings to blend the stimuli into the background. All other settings and procedures are stayed the same.

### Procedure

Each trial began with a fixation display consisting of fixation crosses (0.111×0.111 deg^2^) and high-contrast frames (width: 0.111 deg; length: 6 deg) with diagonal bars (width: 0.111 deg; length: 2.33 deg) in both eyes. The high-contrast frames remained on the screen during the entire experiment to assist observers to fuse the images from the two eyes. After achieving correct fusion, the observer pressed the space bar on the computer keyboard to initiate the presentation of the three sine-wave gratings: two test gratings on the left side of fixation, and a probe grating on the right side of fixation, with the initial contrast and phase of the probe grating set randomly. Observers were required to adjust the contrast and phase of the probe grating to match those of the cyclopean image on the left. They were free to select which dimension to adjust first and to go back and forth on them, and pressed the ‘Enter’ key twice to report the results after they were satisfied with the match in both dimensions. Inter-trial interval of 1 second was provided. A typical trial lasted about 10 seconds.

### Design

We measured the perceived phase and contrast of the cyclopean sine-wave gratings as a function of the base contrast level, the contrast ratio between the two eyes, the phase difference between the sine-wave gratings, and stimulus configurations. Two stimulus configurations were used to cancel potential positional biases [24], [27]: a) left eye phase shift  = Θ/2, right eye phase shift  = −Θ/2, and (b) left eye phase shift  = −Θ/2, right eye phase shift  = Θ/2. Following Ding and Sperling [24], we scored the perceived phase of the cyclopean sine-wave grating as the difference between the measurements from the two configurations. Only the perceived contrast was measured in the in-phase (Θ = 0) condition. There were therefore a total of 216 (3 base contrast levels ×6 interocular ratios×3 phase differences ×2 probe eye conditions×2 configurations) and 144 (3×6×2×2×2) conditions for perceived contrast and phase, respectively.

Each experimental session consisted of one measurement in all experimental conditions, lasting 40 to 90 minutes. The measurements were repeated at least 8 times in separate days. Voluntary breaks were allowed. Practice trials were provided prior to data collection.

### Data fitting procedure

All the model-fitting procedures were implemented in Matlab using a non-linear least-square method that minimized 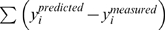, where 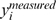 and 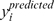 denote measured values and the corresponding model predictions, respectively. The goodness-of-fit was evaluated by the *r* ^2^ statistic for phase and contrast separately:



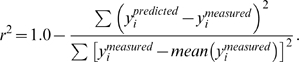
(4)


An F-test for nested models was used to statistically compare the models based on the *r* ^2’^s of phase and contrast. For two nested models with 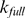 and 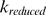 parameters, the 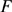 statistic is defined as:
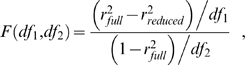
(5)where 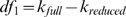, and 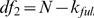; 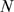 is the number of data points.

## Supporting Information

Text S1 Multipathway ContrastGain Control Model MCM of Binocular Combination.
(DOC)
