# Supplementary material for: Contrast and Phase Combination in Binocular Vision
Source: PLoS One. 2010 Dec 9;5(12):e15075. doi: 10.1371/journal.pone.0015075 (PMC3000330; doi:10.1371/journal.pone.0015075)
Supplement: Text S1 — Multipathway ContrastGain Control Model MCM of Binocular Combination. (DOC) [file pone.0015075.s001.doc]

**Text S1. Multi-pathway Contrast-Gain Control Model (MCM) of Binocular Combination**

The multi-pathway contrast-gain control model (MCM) of binocular combination elaborates the Ding-Sperling binocular combination model (24) in two ways: (1) The perceived phase and contrast of the cyclopean images are computed in separate pathways, although with shared cross-eye contrast-gain control; and (2) phase-independent local energy from the two monocular images are used in contrast combination. We briefly describe the MCM in this supplement.

The input signals for binocular combination, the two monocular sinewave gratings, are defined as:

$Lum_{L}(y)=L_{0}[1-C_{0}\cos(2\pi fy\pm\frac{\theta}{2})]$, (S1)

$Lum_{R}(y)=L_{0}[1-\delta C_{0}\cos(2\pi fy\mp\frac{\theta}{2})]$ (S2)

where $L_{0}$ is the background luminance, $f$ is the spatial frequency of the gratings, $C_{0}$ is the base contrast, and $\delta$ is the interocular contrast ratio. The two gratings are phase-shifted in opposite directions by $\frac{\theta}{2}$, with a total phase difference of $\theta$.

They first go through double interocular contrast gain control (24), in which each eye exerts gain control on the other eye’s signal in proportion to its own signal contrast energy, and also gain-controls over the other eye’s gain control. The signals in the left and right eyes become:

$Lum_{L}^{'}=\frac{1}{1+\frac{\varepsilon_{R}}{1+\varepsilon_{L}}}Lum_{L}$, (S3)

$Lum_{R}^{©}=\frac{1}{1+\frac{\varepsilon_{L}}{1+\varepsilon_{R}}}Lum_{R}$, (S4)

where $\varepsilon_{L}$ and $\varepsilon_{R}$ are the total contrast energy presented to the two eyes and are modelled as $\varepsilon_{L}=\rho C_{L}^{\gamma_{1}}$and $\varepsilon_{R}=\rho C_{R}^{\gamma_{1}}$, ρ is the gain-control efficiency of the signal sine-wave grating, and$\gamma_{1}$ is the exponent of the non-linear transducer. In the experiment, we set *C_L_*=*C_0_* and *C_R_*=δ*C_0_*. From Eqs. S3 and S4, we have:

$Lum_{L}^{'}=\frac{1+\rho C_{0}^{\gamma_{1}}}{1+\rho C_{0}^{\gamma_{1}}+\rho\delta^{\gamma_{1}}C_{0}^{\gamma_{1}}}Lum_{L}$, (S5)

$Lum_{R}^{©}=\frac{1+\rho\delta^{\gamma_{1}}C_{0}^{\gamma_{1}}}{1+\rho C_{0}^{\gamma_{1}}+\rho\delta^{\gamma_{1}}C_{0}^{\gamma_{1}}}Lum_{R}$. (S6)

Following Ding and Sperling (24), the cyclopean image is computed directly from the sum of $Lum_{L}^{'}$ and $Lum_{R}^{'}$:

$Lum^{'}=Lum_{L}^{'}+Lum_{R}^{'}$ (S7)

Substituting Eqs. S1, S2, S5 and S6 into S7, we obtain:

(S8)

Two phase shift conditions are used in our experiment. When the phase is $\frac{\theta}{2}$ in the left eye and $-\frac{\theta}{2}$in the right eye, Eq. S8 can be re-written as:

$Lum^{©}=[\frac{2+\rho C_{0}^{\gamma_{1}}+\rho\delta^{\gamma_{1}}C_{0}^{\gamma_{1}}}{1+\rho C_{0}^{\gamma_{1}}+\rho\delta^{\gamma_{1}}C_{0}^{\gamma_{1}}}-C_{DS1}^{©}\times\cos(2\pi fy+\theta_{DS1}^{©})]L_{0},$ (S9)

with

$C_{DS1}^{©}=\sqrt{(\frac{C_{0}+\rho C_{0}^{1+\gamma_{1}}}{1+\rho C_{0}^{\gamma_{1}}+\rho\delta^{\gamma_{1}}C_{0}^{\gamma_{1}}})^{2}+(\frac{\delta C_{0}+\rho\delta^{1+\gamma_{1}}C_{0}^{1+\gamma_{1}}}{1+\rho C_{0}^{\gamma_{1}}+\rho\delta^{\gamma_{1}}C_{0}^{\gamma_{1}}})^{2}+2\frac{(C_{0}+\rho C_{0}^{1+\gamma_{1}})\times(\delta C_{0}+\rho\delta^{1+\gamma_{1}}C_{0}^{1+\gamma_{1}})}{(1+\rho C_{0}^{\gamma_{1}}+\rho\delta^{\gamma_{1}}C_{0}^{\gamma_{1}})^{2}}\cos\theta}$, (S10)

$\theta_{DS1}^{©}=\tan^{-1}[\frac{1+\rho C_{0}^{\gamma_{1}}-\delta+\rho\delta^{1+\gamma_{1}}C_{0}^{\gamma_{1}}}{1+\rho C_{0}^{\gamma_{1}}-\delta+\rho\delta^{1+\gamma_{1}}C_{0}^{\gamma_{1}}}\tan(\frac{\theta}{2})].$ (S11)

Here we use the subscript “DS” to indicate derivations from the original Ding-Sperling model (24) and thus separate them from the elaborated MCM model.

If the phase is $-\frac{\theta}{2}$ in the left eye and $\frac{\theta}{2}$ in the right eye, Eq S8 can be re-written as:

$Lum^{©}=[\frac{2+\rho C_{0}^{\gamma_{1}}+\rho\delta^{\gamma_{1}}C_{0}^{\gamma_{1}}}{1+\rho C_{0}^{\gamma_{1}}+\rho\delta^{\gamma_{1}}C_{0}^{\gamma_{1}}}-C_{DS2}^{©}\times\cos(2\pi fy+\theta_{DS2}^{©})]L_{0,}$ (S12)

with

$C_{DS2}^{©}=\sqrt{(\frac{C_{0}+\rho C_{0}^{1+\gamma_{1}}}{1+\rho C_{0}^{\gamma_{1}}+\rho\delta^{\gamma_{1}}C_{0}^{\gamma_{1}}})^{2}+(\frac{\delta C_{0}+\rho\delta^{1+\gamma_{1}}C_{0}^{1+\gamma_{1}}}{1+\rho C_{0}^{\gamma_{1}}+\rho\delta^{\gamma_{1}}C_{0}^{\gamma_{1}}})^{2}+2\frac{(C_{0}+\rho C_{0}^{1+\gamma_{1}})\times(\delta C_{0}+\rho\delta^{1+\gamma_{1}}C_{0}^{1+\gamma_{1}})}{(1+\rho C_{0}^{\gamma_{1}}+\rho\delta^{\gamma_{1}}C_{0}^{\gamma_{1}})^{2}}\cos\theta}$, (S13)

$\theta_{DS2}^{©}=-\tan^{-1}[\frac{1+\rho C_{0}^{\gamma_{1}}-\delta+\rho\delta^{1+\gamma_{1}}C_{0}^{\gamma_{1}}}{1+\rho C_{0}^{\gamma_{1}}-\delta+\rho\delta^{1+\gamma_{1}}C_{0}^{\gamma_{1}}}\tan(\frac{\theta}{2})].$ (S14)

To account for possible positional effect, we report the perceived contrast and phase of the cyclopean image as:

$\begin{matrix} C_{DS}^{©}=\frac{C_{DS1}^{©}+C_{DS2}^{©}}{2} \\ =\sqrt{(\frac{C_{0}+\rho C_{0}^{1+\gamma_{1}}}{1+\rho C_{0}^{\gamma_{1}}+\rho\delta^{\gamma_{1}}C_{0}^{\gamma_{1}}})^{2}+(\frac{\delta C_{0}+\rho\delta^{1+\gamma_{1}}C_{0}^{1+\gamma_{1}}}{1+\rho C_{0}^{\gamma_{1}}+\rho\delta^{\gamma_{1}}C_{0}^{\gamma_{1}}})^{2}+2\frac{(C_{0}+\rho C_{0}^{1+\gamma_{1}})\times(\delta C_{0}+\rho\delta^{1+\gamma_{1}}C_{0}^{1+\gamma_{1}})}{(1+\rho C_{0}^{\gamma_{1}}+\rho\delta^{\gamma_{1}}C_{0}^{\gamma_{1}})^{2}}\cos\theta} \end{matrix}$, (S15)

$\begin{matrix} \theta_{DS}^{©}=\theta_{DS1}^{©}-\theta_{DS2}^{©} \\ =2\tan^{-1}[\frac{1+\rho C_{0}^{\gamma_{1}}-\delta+\rho\delta^{1+\gamma_{1}}C_{0}^{\gamma_{1}}}{1+\rho C_{0}^{\gamma_{1}}-\delta+\rho\delta^{1+\gamma_{1}}C_{0}^{\gamma_{1}}}\tan(\frac{\theta}{2})] \end{matrix}$. (S16)

Eq. S15 is clearly inconsistent with our observation that the perceived contrast of the cyclopean images is independent of the relative phase of the two monocular sine-wave gratings. Instead, a phase independent computation is required to model our empirical results. We added an independent contrast pathway to the Ding-Spering binocular combination model. In this pathway, the phase information in$Lum_{L}^{'}$ and $Lum_{R}^{'}$ is discarded. Following interocular contrast gain control, contrast energies in the two eyes are extracted and combined to predict the perceived contrast of the cyclopean image:

$C^{©}=[(\frac{C_{0}+\rho C_{0}^{1+\gamma_{1}}}{1+\rho C_{0}^{\gamma_{1}}+\rho\delta^{\gamma_{1}}C_{0}^{\gamma_{1}}})^{\gamma_{2}}+(\frac{\delta C_{0}+\rho\delta^{1+\gamma_{1}}C_{0}^{1+\gamma_{1}}}{1+\rho C_{0}^{\gamma_{1}}+\rho\delta^{\gamma_{1}}C_{0}^{\gamma_{1}}})^{^{\gamma_{2}}}]^{1/\gamma_{2}}$. (S17)

The computation in the phase pathway is the same as that of the original Ding-Sperling model (24). The perceived phase difference between the two experimental configurations is:

$\theta^{©}=2\tan^{-1}[\frac{1+\rho C_{0}^{\gamma_{1}}-\delta+\rho\delta^{1+\gamma_{1}}C_{0}^{\gamma_{1}}}{1+\rho C_{0}^{\gamma_{1}}-\delta+\rho\delta^{1+\gamma_{1}}C_{0}^{\gamma_{1}}}\tan(\frac{\theta}{2})].$ (S18)

Together, three free parameters, ρ, γ_1_ and γ_2_, are used to model the perceived phase and contrast of the cyclopean images in binocular combination.
